# Rapid generation of functional hepatocyte-like cells from human adipose-derived stem cells

**DOI:** 10.1186/s13287-016-0364-6

**Published:** 2016-08-05

**Authors:** Yanli Fu, Jie Deng, Qingyuan Jiang, Yuan Wang, Yujing Zhang, Yunqi Yao, Fuyi Cheng, Xiaolei Chen, Fen Xu, Meijuan Huang, Yang Yang, Shuang Zhang, Dechao Yu, Robert Chunhua Zhao, Yuquan Wei, Hongxin Deng

**Affiliations:** 1 State Key Laboratory of Biotherapy and Cancer Center/Collaborative Innovation Center of Biotherapy, West China Hospital, Sichuan University, Ke-yuan Road 4, No. 1, Gao-peng Street, Chengdu, Sichuan 610041 People’s Republic of China; 2 Department of Obstetrics, Sichuan Provincial Hospital For Women and Children, Chengdu, People’s Republic of China; 3 Department of Thoracic Oncology, Cancer Center, West China Hospital, Sichuan University, Chengdu, People’s Republic of China; 4 Center of Excellence in Tissue Engineering, Key Laboratory of Beijing, Institute of Basic Medical Sciences and School of Basic Medicine, Chinese Academy of Medical Sciences and Peking Union Medical College, Beijing, People’s Republic of China

**Keywords:** Human adipose derived stem cells, Hepatogenic differentiation, Acute fulminant liver failure, Liver regeneration

## Abstract

**Background:**

Liver disease is a major cause of death worldwide. Orthotropic liver transplantation (OLT) represents the only effective treatment for patients with liver failure, but the increasing demand for organs is unfortunately so great that its application is limited. Hepatocyte transplantation is a promising alternative to OLT for the treatment of some liver-based metabolic disorders or acute liver failure. Unfortunately, the lack of donor livers also makes it difficult to obtain enough viable hepatocytes for hepatocyte-based therapies. Currently, a fundamental solution to this key problem is still lacking. Here we show a novel non-transgenic protocol that facilitates the rapid generation of functional induced hepatocytes (iHeps) from human adipose-derived stem cells (hADSCs), providing a source of available cells for autologous hepatocytes to treat liver disease.

**Methods:**

We used collagenase digestion to isolate hADSCs. The surface marker was detected by flow cytometry. The multipotential differentiation potency was detected by induction into adipocytes, osteocytes, and chondrocytes. Passage 3–7 hADSCs were induced into iHeps using an induction culture system composed of small molecule compounds and cell factors.

**Results:**

Primary cultured hADSCs presented a fusiform or polygon appearance that became fibroblast-like after passage 3. More than 95 % of the cells expressed the mesenchymal cell markers CD29, CD44, CD166, CD105, and CD90. hADSCs possessed multipotential differentiation towards adipocytes, osteocytes, and chondrocytes. We rapidly induced hADSCs into iHeps within 10 days in vitro; the cellular morphology changed from fusiform to close-connected cubiform, which was similar to hepatocytes. After induction, most of the iHeps co-expressed albumin and alpha-1 antitrypsin; they also expressed mature hepatocyte special genes and achieved the basic functions of hepatocyte. Moreover, iHep transplantation could improve the liver function of acute liver-injured NPG mice and prolong life.

**Conclusions:**

We isolated highly purified hADSCs and rapidly induced them into functional hepatocyte-like cells within 10 days. These results provide a source of available cells for autologous hepatocytes to treat liver disease.

## Background

The liver is the largest internal organ in the human body and the main site of drug metabolism and its resulting toxicity. Liver diseases are crucial causes of death worldwide. Orthotropic liver transplantation (OLT) represents the only effective treatment for patients with liver failure, but the increasing demand for organs is unfortunately so great that many patients die while awaiting transplantation [1]. Hepatocyte transplantation has been proposed as an alternative to whole-organ transplantation to support many forms of hepatic insufficiency. Unfortunately, the lack of donor livers also makes it difficult to obtain enough viable hepatocytes for hepatocyte-based therapies. To solve this dilemma, novel strategies for generating ample hepatocytes are in high demand.

Mesenchymal stem cells (MSCs) have become a prominent resource for regenerative medicine as readily available cells, which can be obtained from different sources such as bone marrow [2], umbilical cord blood [3], amniotic fluid [4], scalp tissue [5], placenta [6], or adipose tissue [7] of the human body. These cells show a multipotentiality and semi-infinite proliferation ability. In particular, adipose-derived stem cells (ADSCs) are recognized as one of the most promising MSCs identified thus far since adipose tissue is ubiquitous and easily obtained in large quantities with little donor site morbidity or patient discomfort [8–13]. Furthermore, recent research has revealed that the use of ADSCs in regenerative medicine is not limited to mesodermal tissue but extends to both ectodermal and endodermal tissues and organs, although ADSCs originate from mesodermal lineages [14, 15].

Here, we describe the isolation of MSCs from human abdominal adipose tissue and their differentiation into hepatocyte-like cells (induced hepatocytes (iHeps)) in vitro using selective cell factors and small molecule compounds. Post-transplantation of hepatocyte-like cells differentiated from human adipose derived stem cells (hADSCs) in experimental models of hepatic injury by carbon tetrachloride could express hepatic specific genes, and possess the functional properties of mature hepatocytes, including secretion of albumin, synthesis of albumin (ALB) and cytochrome P450 enzyme activity. iHeps could restore the liver function and prolong survival. The results show a very important step towards future establishment of an alternative and successful therapy for autologous hepatocytes to treat liver disease.

## Methods

### Isolation, culture, and expansion of hADSCs

hADSCs were isolated from human female abdominal adipose tissue obtained during Caesarean section. The adipose tissue was washed with Hank’s balanced salt solution (HBSS; Gibco). The washing step was repeated until all blood vessels and connective tissues appeared to have been liberated (usually three washes). The adipose tissue sample was minced into small pieces and digested in Dulbecco’s modified Eagle’s medium LG (DMEM-LG; Gibco) containing 1 mg/mL (w/w) collagenase Type I, and then incubated at 37 °C for 30 min with gentle shaking at 120 rpm. After the addition of fetal bovine serum (FBS; Gibco) to a final concentration of 10 % (v/v), the solution was filtered through a 100-μm filter (BD Falcon) to remove solid aggregates. The sample was subsequently centrifuged at 1500 rpm for 5 min at 4 °C. The pellet was washed twice with ice-cold HBSS and centrifuged at 1500 rpm for 5 min. The supernatant was removed and the cell pellet was resuspended in complete medium (DMEM-LG with 15 % FBS and 1 % antibiotic antimycotic solution) in a 75-cm^2^ culture dish and maintained in an incubator supplied with a humidified atmosphere of 5 % CO_2_ at 37 °C. After 2 days, non-adherent cells were removed by two or three washes with HBSS and adherent cells were further cultured in complete medium. The medium was changed every 2 days until the monolayer of adherent cells reached 80–90 % confluence. Cell passaging was performed using a 0.25 % trypsin solution (Sigma-Aldrich). Approximately 3 × 10^5^ cells were used to inoculate a 75-cm^2^ culture dish and incubated at 37 °C and 5 % CO_2_.

### Primary human hepatocyte isolation

Liver specimens were obtained from the margin of the macroscopically tumor-free liver tissue immediately after resection. Samples were taken under sterile conditions, transferred into ice cold (4 °C) William’s E medium, and human primary hepatocytes were immediately isolated under sterile conditions using collagenase. The liver specimen was washed twice with ice-cold HBSS. The washing step was repeated until all blood vessels and connective tissues appeared to have been liberated (usually three washes). The specimens were then cut into small pieces and digested with William’s E medium containing IV collagenase, and incubated at 37 °C for 30 min with gentle shaking at 120 rpm. After the completion of digestion, a final concentration of 10 % (v/v) FBS was added to stop the collagenase reaction. The resulting cell suspension was filtered through a 100-μm filter (BD Falcon) for the removal of solid aggregates. The filtered sample was subsequently centrifuged at 1500 rpm for 5 min at 4 °C. Finally, the cell pellets were washed with HBSS followed by another centrifugation (1500 rpm for 5 min at 4 °C).

### Flow cytometry analysis

For flow cytometry detection of surface antigens, hADSCs (1 × 10^6^ cells) were washed and resuspended in stain buffer (PBS; BD Biosciences) containing saturating concentrations (1:100 dilution) of the following conjugated mouse or rat monoclonal antibodies against human antigens (Biolegend) for 30 min in the dark at 2–8 °C: CD105-FITC, CD90-FITC, CD44-FITC, CD31-APC, CD34-PE, CD29-PE, CD45-PerCP/cy5.5, CD166-PE, HLA-DR-PE and FITC-CD90, CD105 labeled mouse IgG1k Isotype control, FITC-CD44 labeled Rat IgG2b k Isotype control, PerCP/cy5.5-CD45 labeled mouse IgG1 k Isotype control, APC-CD31 labeled mouse IgG1 k Isotype control, PE-CD29, 166 labeled mouse IgG1 k Isotype control, and PE-HLA-DR labeled Mouse IgG2a k Isotype control.

For intracellular staining of ALB and alpha-1-antitrypsin (AAT), 5 × 10^5^ iHeps were harvested and fixed with 4 % paraformaldehyde for 30 min, and then permeabilized in staining buffer (BD) for 10 min. Cells were then incubated with primary antibody (Goat anti-human Albumin, Bethyl; Mouse anti-human AAT, Thermo) in staining buffer overnight at 4 °C, followed by secondary antibody (dylight 488 conjugated Donkey anti-goat IgG, Bethyl; dylight 594 conjugated Donkey anti-mouse IgG, Bethyl) incubation for 1 h at 37 °C. The cell suspensions were washed twice and resuspended in 300–500 μL PBS for flow cytometry (FACS Aria, BD Biosciences) using FLOWJO TM software (TreeStar, Inc., Ashland, OR, USA).

### Adipogenic differentiation

Passage 3 hADSCs were counted and seeded at a density of 10^5^ per well in a six-well plate. When the cells reached 100 % confluence, Adipogenic Differentiation Basal Medium A supplemented with 10 % FBS, 1 % penicillin-streptomycin, dexamethasone, isobutylmethylxanthine (IBMX), insulin, glutamine, and Rosiglitazone (Cyagen Biosciences) was added to four wells and complete culture medium (DMEM-LG) was added to other two wells as the negative controls. Three days later the medium was changed to hADSC Adipogenic Differentiation Basal Medium B, which contains 10 % FBS, dexamethasone, insulin, and glutamine (Cyagen Biosciences). The medium was changed 24 h later to medium A. Medium A and B were alternated 3–5 times (12–20 days), and then medium B was maintained for 4–7 days until the lipid droplets were big and round enough. During maintained culture, medium B was changed every 2–3 days with fresh medium B. Oil red O staining assessed the differentiation potential of adipogenesis formation of intracellular lipid droplets.

### Osteogenic differentiation

Passage 3 hADSCs were harvested by trypsin digestion as described above; the cells were counted and seeded at a density of 10^5^ per well in a six-well plate. When the cells reached 100 % confluence, MSC Osteogenic Differentiation Basal Medium containing 10 % FBS, 1 % penicillin-streptomycin, glutamine, ascorbate, β-glycerophosphate, and dexamethasone (Cyagen Biosciences) was added to four wells while complete culture medium was added to the other two wells as the negative controls. The medium was changed every 3 days for 3 weeks. The differentiation potential for osteogenesis was assessed by 40 mM Alizarin Red (pH 4.2) staining.

### Chondrogenic differentiation

Passage 3 hADSCs were counted and seeded at a density of 10^6^ per well in an ultra-low attachment six-well plate. When the pellet cultures contained 2.5 × 10^5^ hADSCs, MSC Chondrogenic Differentiation Basal Medium, which consists of dexamethasone, ascorbate, sodium pyruvate, proline, TGF-β3, and insulin-transferrin-selenium (ITS; Cyagen Biosciences), was added to four wells and complete culture medium was added to the other two well as the negative controls. The medium was replaced every 3 days for 3 weeks. The differentiation potential for chondrogenesis was measured by Alcian blue staining.

### Hepatic differentiation of hADSCs in vitro

hADSCs between passage 3 and 7 were planted at a density of 2–3 × 10^4^ cells/cm^2^ on collagen I-coated dishes (Invitrogen) and cultured in expansion medium at 37 °C with 5 % CO_2_. Once the cells reached 100% confluence, they were incubated with 10 % FBS RPMI-1640 (Gibco) medium containing 1 μM ATRA for 24 h. The cells were then incubated with serum-free RPMI-1640 medium containing 100 nM IDE1, 3 μM CHIR99021, and 10 μM LY294002 (Selleckchem) for 24 h. Next, the cells were then incubated with serum-free RPMI-1640 medium containing 100 nM IDE1, 10 μM LY294002, 250 nM LDN-193189, and 20 ng/mL FGF4 (PeproTech) for 2 days and then changed to serum-free RPMI-1640 medium containing 100 nM IDE1, 10 μM LY294002, and 20 ng/mL FGF4 for 24 h. The medium was changed to Williams’ E (Gibco) supplemented with 150 ng/mL hepatocyte growth factor (HGF; Sino Biological), 20 ng/mL FGF4, 30 ng/mL oncostatin M (OsM; PeproTech), 2 × 10^−5^ mol/L dexamethasone (Dex, Sigma-Aldrich), and 1% ITS (Sigma-Aldrich). The differentiation medium was changed every 2 days.

### Immunofluorescence

For immunofluorescent staining, the cells were fixed with 4 % paraformaldehyde for 15 min at room temperature, and then incubated with PBS containing 0.1 % Triton X-100 (Sigma-Aldrich) for 15 min. Cells were then washed three times with PBS. After being blocked by 5 % BSA in PBS for 1 h at room temperature, cells were incubated with primary antibodies at 4 °C overnight, washed three times with PBS, and then incubated with appropriate fluorescence-conjugated secondary antibody for 1 h at room temperature in the dark. Nuclei were stained with DAPI (Sigma-Aldrich). Primary and secondary antibodies were diluted in PBS containing 3 % BSA. Antibodies used for immunofluorescence were as follows: Goat anti-human Albumin (Bethyl, 1:200), Mouse anti-human AAT (Thermo, 1:40), dylight 488 conjugated Donkey anti-goat IgG (Bethyl, 1:200), dylight 594 conjugated Donkey anti-mouse IgG (Bethyl, 1:200), Rabbit anti-human AFP (Santa, 1:500), Mouse anti-human FOXA2 (Abcam, 1:250), Mouse anti-human hepatocyte (HepPar1) (Gene Tech, 1:20).

### Reverse transcription-polymerase chain reaction (RT-PCR)

RNA (1 μg) was reverse transcribed into cDNA with the M-MLV Reverse Transcriptase (Promega) according to the manufacturer’s instructions. PCR was performed with HiFi Taq polymerase (TransGen). On completion of the PCR, products were examined on 1 % agarose gel. β-actin primers were used as an internal standard and amplifications of products were performed at 30 cycles. Primer sequences are provided in Table 1.

**Table 1:** Forward (F) and reverse (R) primer pairs used for polymerase chain reactions to detect hepatic specific gene transcripts in human adipose-derived stem cells, induced heaptocytes, and primary human hepatocytes

Primer	Sequence (5′–3′)
AAT	F: TGAGTTCGCCTTCAGCCTATACC
	R: AGTCCTCTTCCTCGGTGTCCTT
ALB	F: GGTGAGACCAGAGGTTGATGTG
	R: GCAGCAGCACGACAGAGTAATC
AFP	F: GCAGCCAAAGTGAAGAGGGAAGAC
	R: GCAGACAATCCAGCACATCTCCTC
HNF4α	F: GGTGTCCATACGCATCCTTG
	R: TGTCCGTTGCTGAGGTGAG
TDO2	F: CCGTAGAAGGCAGCGAAGAAG
	R: GCTCCCTGAAGTGCTCTGTATG
CYP3A4	F: ATGGTCAACAGCCTGTGCT
	R: CATGCTGTAGGCCCCAAAGA
TTR	F: GGCATCTCCCCATTCCATGA
	R: TTCCTTGGGATTGGTGACGAC
CK18	F: ATGAGCTTCACCACTCGCTC
	R: TGGCAATCTGGGCTTGTAGG
CK19	F: GCTTTGTGTCCTCGTCCTCC
	R: TTGGCTTCGCATGTCACTCA
CK7	F: CCCAGACATCTTTGAGGCCC
	R: TTCACGGCTCCCACTCCATC
human ALU	F: AATATGGCCCAACTGCAGAA
	R: CATCGCATTTTCACATCCAA
β-actin	F: GGCATCGTGATGGACTCCG
	R: GCTGGAAGGTGGACAGCGA

### Real-time quantitative PCR

Total RNA was isolated from hADSCs, iHeps, and primary human hepatocytes (PHH) using the Trizol Reagent (Sigma-Aldrich). Quantitative real-time PCR was performed with SYBR Premix Ex Taq (TaKaRa) on the ABI StepOnePlus real-time PCR system (Applied Biosystems). All quantitative PCR data were obtained with at least two repeats. The PCR products were confirmed by proper melting curves. β-actin primers were used as an internal standard and amplifications of products were performed at 39 cycles. Primer sequences are provided in Table 2.

**Table 2:** Forward (F) and reverse (R) primer pairs used for polymerase chain reactions to detect hepatic specific gene transcripts in human adipose-derived stem cells, induced heaptocytes, and primary human hepatocytes

Primer	Sequence (5′–3′)
β-actin	F: TGGACTTCGAGCAAGAGATG
	R: GAAGGAAGGCTGGAAGAGTG
CYP1A2	F: CTTCGCTACCTGCCTAACCC
	R: GACTGTGTCAAATCCTGCTCC
CYP1A1	F: CAAGGGGCGTTGTGTCTTTG
	R: GTCGATAGCACCATCAGGGG
CYP2A6	F: CAGCACTTCCTGAATGAG
	R: AGGTGACTGGGAGGACTTGAGGC
CYP2B6	F: GCACTCCTCACAGGACTCTTG
	R: CCCAGGTGTACCGTGAAGAC
CYP2C9	F: CTACAGATAGGTATTAAGGACA
	R: GCTTCATATCCATGCAGCACCAC
CYP2D6	F: TGAAGGATGAGGCCGTCTGGGAGA
	R: CAGTGGGCACCGAGAAGCTGAAGT
CYP3A4	F: TTCAGCAAGAAGAACAAGGACAA
	R: GGTTGAAGAAGTCCTCCTAAGC

### Western blot analysis

Western blotting was used to detect the presence of CK18, HNF4α, AFP, ALB, and AAT in iHeps. The cells were dissolved in the Mammalian Protein Extraction Reagent (Pierce, Rockford, IL, USA). Proteins were separated on an SDS-polyacrylamide gel and transferred to PVDF (polyvinylidine difluoride) membranes (BIORAD, Tokyo, Japan). Blots were saturated with 5 % skimmed milk in TBS-T for 1 h at room temperature, and afterwards incubated overnight with anti-human mouse monoclonal CK18 (Santa Cruz Biotechnology, Inc., CA, USA), rabbit polyclonal HNF4α (Santa Cruz Biotechnology, Inc.), rabbit polyclonal AFP (Santa Cruz Biotechnology, Inc.), mouse polyclonal ALB (Santa Cruz Biotechnology, Inc.), and rabbit polyclonal AAT (Abcam). Following washing in TBS-T, the membranes were incubated for 30 min with sheep anti-rabbit or anti-mouse IgG-HRP-linked whole antibodies (GE Healthcare Bio-Sciences KK, Tokyo, Japan). Monoclonal antibodies against human GAPDH were used as a control of protein loading (Protein Tech).

### Periodic acid-schiff (PAS) staining

After 4 % paraformaldehyde fixation, cells were incubated for 5 min in 1 % periodic acid (Sigma, St. Louis, MO, USA) and washed with distilled water prior to incubation with Schiff’s reagent (Sigma) for 15 min. After a 5-min wash in tap water, cells were washed and visualized under a light microscope (CKX41; Olympus, Japan).

### Indocyanine green and Oil Red O staining

For indocyanine green (ICG; Sigma-Aldrich) uptake assay, cells were changed to a medium with 1 mg/mL ICG and incubated at 37 °C for 1 h, followed by washing with PBS three times. For Oil red O staining, confluent cells were cultured in hepatic differentiation medium. After 10 days, cells were washed twice with PBS, fixed in 4 % formalin for 30 min, washed with PBS twice, followed by Oil Red O (Sigma-Aldrich) staining for 10 min, and then washed twice with 70 % ethanol and visualized under a light microscope (CKX41; Olympus).

### Uptake of low-density lipoprotein assay

The uptake of low-density lipoprotein (LDL) was detected with the Dil-Ac-LDL staining kit (Biomedical Technologies, Stoughton, MA, USA). The assay was performed according to the manufacturer’s instructions. Briefly, cells were incubated in serum-free DMEM-LG containing 10 μg/mL 1,1′-dioctadecyl-3,3,3′,3′-tetramethy-lindocarbocyanine perchlorate acetylated-LDL (Dil-Ac-LDL) for 4 h at 37 °C. Cells were then washed and visualized under a fluorescence microscope (BX51; Olympus).

### Albumin and alpha-1-antitrypsin ELISA

To determine the secretion of human ALB and AAT, supernatants of the cell culture were collected at different time points. For transplantation experiments, animal serum was collected. Levels of human ALB and AAT were measured by the human Albumin ELISA kit (Bethyl Laboratory) and the human α-1-antitrypsin ELISA kit (Bethyl Laboratory) according to the manufacturer’s instructions. Serum was diluted in a range from 10- to 10,000-fold to obtain values falling within the linear range of the standard curve.

### Mice

The genetic background of the NPG mice (Beijing Vitalstar Biotechnology Co., Ltd.) was NOD-Prkdc^scid^IL2rg^null^. For non-obese diabetes (NOD) mice, the phagocytic function of the macrophage for human cells was weak and the innate immune systems, such as the complement system and dendritic cell function, was significantly decreased. The Prkdc^scid^:Prkdc (protein kinase DNA-activated catalytic) gene mutation brings about the loss of T cells and B cells, which causes the severe combined immunodeficiency in both cellular and humoral immunity in mice (severe combined immune deficiency (SCID)). The IL2rg^null^ (interleukin-2 receptor gamma chain (IL-2Rγc), also known as CD132) is the common receptor subunit of cytokines, such as  IL-2, IL-4, IL-7, IL-9, IL-15 and IL-21, which has an important immune function. The mice immune function was severely reduced after knockout of this gene, the activity of natural killer (NK) cells was especially almost lost. All animal experiments were performed in accordance with institutional animal regulations.

### iHep transplantation to CCl_4_-induced acute liver failure mice

NPG mice were injected with CCl_4_ (Sigma-Aldrich) at a dose of 0.5 mL/kg body weight by intraperitoneal injection. Eight hours after CCl_4_ treatment, hADSCs and iHeps (1 × 10^6^ cells/animal, 300 μL) were intravenously injected into the mice. Meanwhile, control animals received an equal volume of PBS. Blood and liver samples were collected after the surviving animals were sacrificed. Blood samples were used for blood biochemical analysis. Livers of recipient mice were embedded in Tissue Freezing Medium (Leica) and then frozen in liquid nitrogen. Cryostat sections (8 μm) were stained.

## Results

### Identification and characterization of hADSCs

**Fig. 1:**
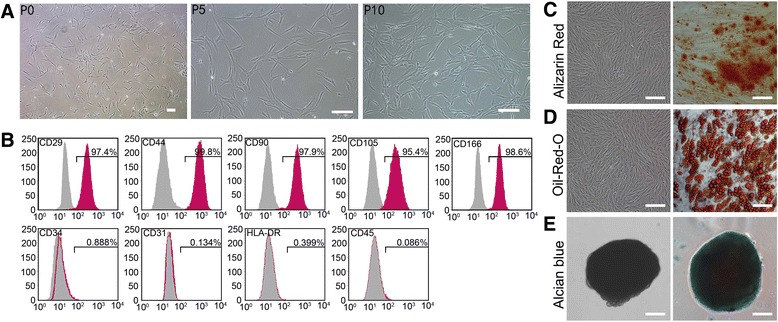
Characterization of human adipose-derived stem cells (hADSCs). **a** Image of primary hADSCs, passage (P)5 hADSCs, and passage 10 hADSCs. The cells showed homogenous fibroblastic morphology. **b** Expression of cell surface markers on hADSCs. hADSCs possessed the surface marker profile typical for mesenchymal stem cells, and were positive for the mesenchymal markers CD29, CD44, CD166, CD105, and CD90, and negative for the hematopoietic and endothelial markers CD45, CD34, and CD31), and showed almost no expression of HLA-DR. **c**–**e** Multiple differentiation potential of hADSCs. Under specific induction conditions, they could differentiate into adipose cells (**d**) osteocytes (**c**), and chondrocytes (**e**). *Scale bars* = 250 μm (**a**, **P0**), 100 μm (a-P5, P10, c, d, e)

The cells isolated from the abdominal fat tissue of healthy women, and the cultures were observed using an inverted light microscope. The attachment of spindle-shaped cells to the culture dish was observed after 1 day of culture. Primary cultures reached 80–90 % confluence in approximately 4–5 days. During the passaging, the cell growth tended to accelerate and the morphology of the cells changed gradually. After three passages, the cultures showed homogenous fibroblastic morphology and, at ten passages, the cell morphology did not change (Fig. 1a). hADSCs expressed the surface marker profile typical for MSCs using flow cytometry. The cells were positive for the mesenchymal markers CD29, CD44, CD166, CD105, and CD90, and negative for hematopoietic and endothelial markers CD34, CD45, and CD31. They also had low immunogenicity, and low or almost no expression of HLA-DR. As shown in Fig. 1b, under adipogenic, osteogenic, and chondrogenic differentiation conditions the cells were able to differentiate into osteocytes, confirmed by Alizarin Red staining (Fig. 1c). They were also able to differentiate into adipose cells, verified by Oil red O staining (Fig. 1d). Furthermore, they could differentiate into chondrocytes, identified by Alcian blue binding assay (Fig. 1e). The results showed that we isolated high purity hADSCs which exhibited typical characteristics of MSCs and had good pluripotent differentiation potential.

### Induction of hADSCs into iHeps in vitro

**Fig. 2:**
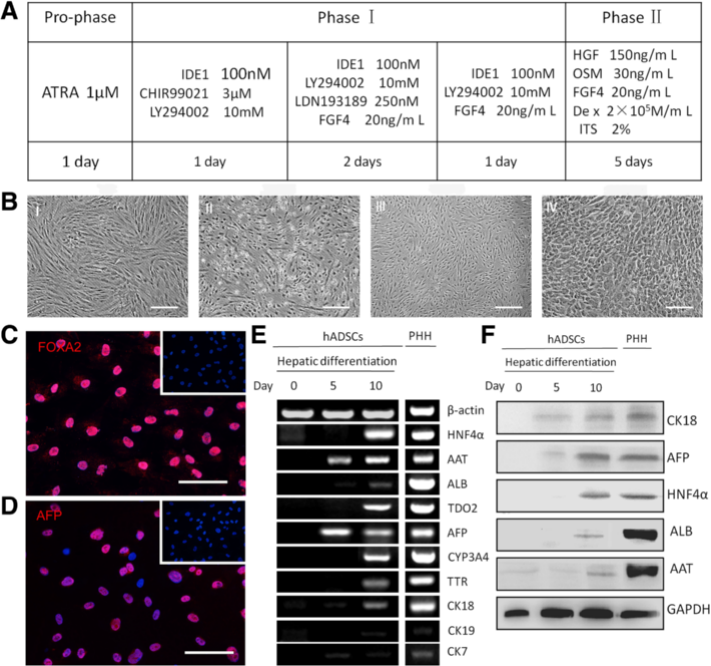
Induction protocols to generate iHeps from human adipose-derived stem cells (*hADSCs*). **a** Schematic diagram of hADSC differentiation into iHeps. **b** The sequential morphological changes from hADSCs to iHeps; I Cell morphology before hepatic induction; II The change of cell morphology with a short spindle shape after 1 day culture in the phase I induction medium; III At day 5, the cell morphology had become polygonal in shape; IV The morphology of hepatocyte-like cells (iHeps). **c**, **d** Immunostaining revealed the vast majority of induced cells were positive for the endoderm marker FoxA2 and early hepatocytes marker AFP at the fifth day (*upper right*: hADSCs). **e** Gradually increased hepatic gene expression and slowly diminishing or disappearing billiard gene expression during the induction of iHeps. The expression levels of the indicated genes were analyzed by RT-PCR. **f** Western blot analysis indicated that C18 (45 kDa), AFP (69 kDa), HNF4a (54 kDa), ALB (66 kDa), and AAT (57 kDa) were actively synthesized in hADSC-derived hepatocytes; GAPDH was used as an internal control (35 kDa). *Scale bars* = 100 µm (**b**) and 25 µm (**c**, **d**). *PHH* primary human hepatocyte; *ATRA* All trans retinoic acid

We applied a three-step protocol (described in Methods) and to induce hepatic differentiation of hADSCs (Fig. 2a). In this protocol, hADSCs were allowed to reach approximately 100 % confluence on collagen type I-coated dishes. The cell morphology is shown in Fig. 2b (panel I). These were then treated with ATRA for 24 h, followed by treating with endodermal induction medium in the presence of IDE1, CHIR99021, and LY294002. After 24 h, the cell morphology had changed into a short spindle shape from that of a long spindle shape (Fig. 2b, panel II). Following the endodermal induction step, cells were treated with the hepatogenic induction medium for 3 days, which changed the cell morphology from a spindle shape to a polygonal shape (Fig. 2b, panel III). Immunofluorescent staining revealed that some of the cells were positive for the endoderm marker FOXA2 (Fig. 2c) and early liver cell marker AFP (Fig. 2d), indicating that the hADSCs differentiated into hepatic precursor cells. Finally, the medium was changed to maturation medium, which resulted in cell morphology changing into the typical cuboidal shape of primary hepatocytes that had tight cell-cell contact (Fig. 2b, panel IV). In brief, over a period of 10 days of culture, cell morphology changed from a spindle to a rather polygonal shape typically associated with adult hepatocytes. Linked with this transformation, the expression of cytokeratin (CK)18, an intermediary filament protein predominantly expressed in epithelial cells, was upregulated as demonstrated by RT-PCR and Western blot analysis (Fig. 2e, f), which showed that hADSCs transformed into epithelial cells from mesenchymal cells. The hepatocytic phenotype of the cells was further substantiated by the appearance of functional in vitro markers barely expressed in undifferentiated cells. By 10 days post-induction, differentiated cells expressed genes specific for mature hepatocytes and these genes increased gradually during hepatic induction, suggesting that hepatogenic differentiation is a progressively coordinated process. In contrast, CK19 and CK7 were upregulated within 5 days of culture, slowly diminishing or disappearing thereafter (Fig. 2e, f). These results indicated that our induction system mainly directed hADSC differentiation into hepatocytes rather than to cholangiocytes. In other words, we had successfully obtained hADSC-derived induced hepatocytes using a new non-transgenic protocol.

### iHeps assumed functional attributes of mature hepatocytes

**Fig. 3:**
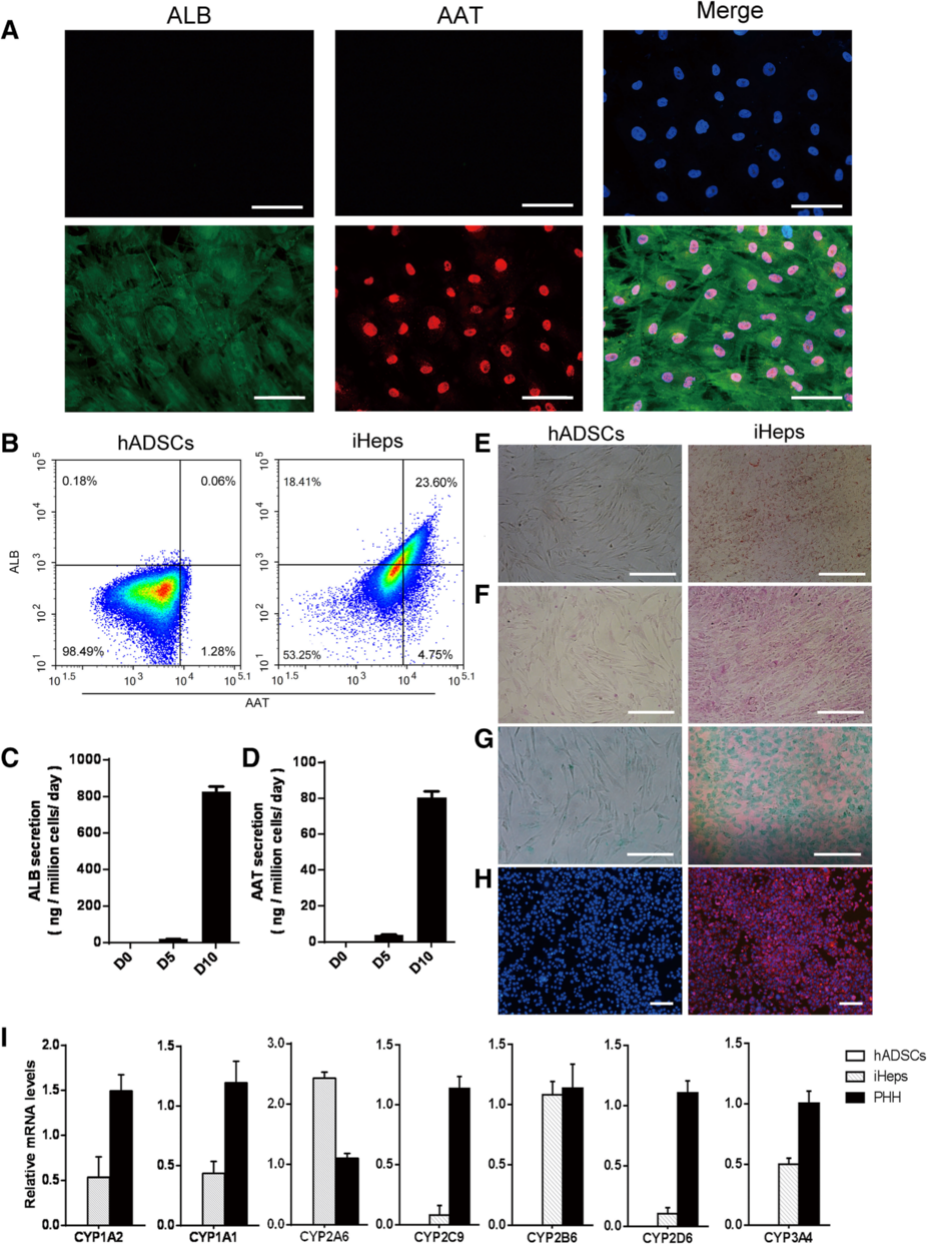
Characterization of induced hepatocytes (*iHeps*) in vitro. **a** Immunofluorescence analysis of albumin (*ALB*) and alpha-1 antitrypsin (*AAT*) in iHeps. Most iHeps expressed both ALB and AAT at the tenth day. **b** iHeps were stained for ALB and AAT at 10 days after induction. ALB and AAT double-positive cells, as quantified by flow cytometry, were used to determine the conversion efficiency of iHeps. **c**, **d** Secretion of ALB and AAT increased during the hepatogenic induction period as measured by ELISA. **e–h** Analysis of basic hepatic function in iHeps, including Oil-Red-O staining (**e**), PAS staining (**f**), ICG intake (**g**), and LDL uptake (**h**). **i** The mRNA levels of CYP genes were determined by qPCR in human adipose-derived stem cells (*hADSCs*), iHeps, and primary human hepatocytes (*PHH*). Data are normalized to β-actin. *Scale bars* = 100 μm (**a, e, f, g**), 50 μm (**h**)

We measured the secretion of human ALB, AAT, glycogen synthesis, Dil-ac-LDL, ICG uptake and the metabolism of lipoid, and CYP enzyme activity to test whether iHeps derived from hADSCs assumed the functional attributes of mature hepatocytes. The ALB and AAT synthesis assay is a specific test for the presence and metabolic activity of mature hepatocytes. Approximately 25 % of cells expressed both ALB and AAT at day 10 as determined by immunofluorescent staining and flow cytometry (Fig. 3a, b), indicating that the iHeps possessed the capacity to produce albumin and alpha-1 antitrypsin. To assess the metabolic activity of iHeps we quantified ALB and AAT secretion. We found that iHeps had a remarkable capacity for secreting the plasma proteins ALB and AAT, which gradually increased in the iHep culture system during the hepatogenic induction period (Fig. 3c, d). iHeps also displayed numerous hallmark functions of mature hepatocytes, such as metabolism of lipoid, glycogen storage, acetylated low-density lipoprotein (ac-LDL) intake, and ICG uptake (Fig. 3e–h). The drug metabolic capacity is one of the most important functions that distinguish hepatocytes. Cytochrome P450 (CYP450) enzymes of hepatocytes are the main enzymes accounting for drug metabolism. Their activity is used to evaluate drug metabolism of hepatocytes. We quantitatively confirmed the expression of the CYP enzymes CYP1A1, CYP1A2, CYP2A6, CYP2C9, CYP2B6, CYP2D6, and CYP3A4 in iHeps. The results showed that iHeps already expressed these genes at remarkable levels (Fig. 3i). These results offer the possibility of using iHeps for toxicity screening during drug discovery.

### Therapy potential of iHeps in CCl_4_-induced acute fulminant liver failure

**Fig. 4:**
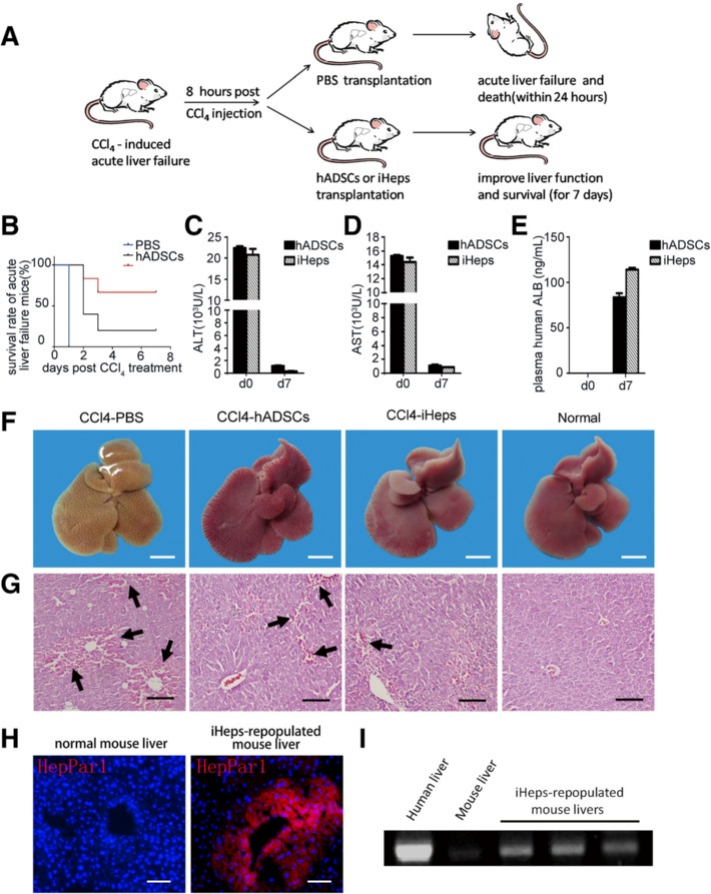
The therapeutic effects of induced hepatocytes (*iHeps*) on acute liver failure. **a** Schematic diagram of cell transplantation into the livers of NPG mice. NPG mice were intraperitoneally injected with carbon tetrachloride (CCl_4_) to trigger fulminant hepatic injury. Eight hours after CCl_4_ treatment, human adipose-derived stem cells (hADSCs) and iHeps (1 × 106 cells/animal, 300μL) were intravenously injected into the mice and control animals received equal volume of phosphate-buffered saline (PBS). **b** Kaplan-Meier survival curve of NPG mice with acute liver failure. **c**, **d** Serum levels of glutamic-pyruvic transaminase (*ALT*) (**c**) and glutamic oxalacetic transaminase (*AST*) (**d**) in CCl_4_-treated mice before (day 0 (*d0*)) and after (*d7*) transplantation of hADSCs or iHeps. **e** Serum levels of human albumin (*ALB*) before (d0) and after (d7) transplantation of hADSCs or iHeps. The albumin antibody is human specific. **f**, **g** Livers in CCl_4_-treated mice 8 h later; PBS, hADSCs, and iHeps were intravenously injected into the mice. Macroscopic images of freshly isolated livers (**f**) and hematoxylin and eosin staining of liver sections (**g**) are shown after treatment. The liver has already recovered from hepatitis by the seventh day. Arrows represent CCl_4_-induced hepatitis and hemorrhage in the liver. **h** The integration of iHeps in mice livers was determined by immunostaining for human HepPar1 in serial sections. **i** Human-specific Alu sequences were analyzed by PCR using genomic DNA extracted from iHep-repopulated mice livers. *Scale bars* = 5 mm (**f**) and 100 μm (**g, h**). Data are presented as mean ± SD

We used serial transplantation to investigate whether iHeps could have sufficient hepatic functions to support the liver in recovering from acute fulminant hepatic injury. NPG mice were injected with CCl_4_ to trigger acute fulminant hepatic injury; 8 h later, intravenous transplantation of either differentiated hepatocytes or undifferentiated hADSCs (1 × 10^6^ cells/animal, 300 μL), or an equal volume of PBS into control animals, was performed (Fig. 4a). The group transplanted with PBS all died within 24 h; 7 days after the injection of CCl_4_, 1 of 5 recipient mice survived after transplantation with hADSCs (survival rate 20 %) and 3 of 5 mice receiving iHep transplantation survived (survival rate 60 %). The Kaplan-Meier survival curve is depicted in Fig. 4b. Transaminase activity was measured and, 7 days after CCl_4_ treatment, the mice receiving iHep transplantation showed nearly normal levels of serum glutamic-pyruvic transaminase (ALT) and glutamic oxalacetic transaminase (AST) (Fig. 4c and d). ELISA analysis of human-specific albumin antibody showed repopulation of iHeps in the liver parenchyma in the surviving mice which could secret human ALB (Fig. 4e). Histological analysis revealed that iHeps significantly improved recovery from CCl_4_-induced liver damage (Fig. 4f, g). Immunofluorescence staining of human HepPar1 showed that iHeps repopulated the liver parenchyma in the surviving mice. Repopulated cells were positively stained by HepPar1, an antibody specifically labeling human hepatocytes, but not mouse hepatocytes or non-hepatic cells (Fig. 4h). Engraftment of iHeps in recipient livers was further confirmed by genomic PCR for human-specific Alu DNA sequences (Fig. 4i), and showed that repopulated iHeps were micro-detected in the NPG mice liver and replaced necrotic hepatocytes to restore liver function. Therefore, we can conclude that, under normal conditions, the surviving mice are capable of long-term survival. Together, this indicates that iHeps may have potential clinical application as a treatment for acute liver injury.

## Discussion

MSCs have great potential for clinical application in regenerative medicine. Adipose tissue is an important source of MSCs. A sufficient number of stem cells (that is, hADSCs) can be easily obtained with minimal invasion from a patient’s own adipose tissue, and hADSCs can be easily isolated and cultured in vitro. hADSCs have a broader differentiation potential and can differentiate into all mesodermal lineage cells, including adipocytes, osteocytes, and chondrocytes. However, they do not express hematopoietic cell or endothelial cell antigens, and also have low immunogenicity. Unfortunately, the subsequent interaction of transplanted undifferentiated ADSCs with liver parenchyma cells in the context of fibrosis, cancer, or liver dysfunction and whether they promote the development of these symptoms is still unknown. Moreover, recent studies demonstrated long-term engraftment of MSC-derived hepatocyte-like cells in a xenogeneic transplantation model of liver regeneration. Engraftment was significantly improved using MSCs pre-differentiated to hepatocyte-like cells in vitro as compared to undifferentiated MSCs [16]. Therefore, we transplanted hepatocyte-like cells differentiated from ADSCs to treat acute fulminant hepatic injury. Several studies have described the differentiation of hADSCs into cells that display hepatic characteristics [16–22]. However, those in vitro differentiation methods are not applicable to a practical and clinical use, as about 1 month is required to induce hADSCs into cells with hepatic functions and using viral vector-integrated TFs (Transcription factors) to reprogram hADSCs into hepatocytes has substantial safety problems. Clinical applications in the future will require a special approach, such as shortening and making as safe as possible ex vivo manipulations, including cultivation and direct hepatic fate. In the present study, we developed a novel non-transgenic protocol that rapidly generated functional hepatocytes from hADSCs within a very short time. Ten days is sufficient to generate in vitro cells which show hepatocyte specific morphology and functionality. To our knowledge, this is the first time such a short hepatogenic differentiation protocol has been presented.

For specific tissue-derived stem cells, such as adipose tissue-derived MSCs, when differentiating into hepatocytes, induction of various tissues isolated from specific stem cells and even embryonic stem cells into specific endodermal cells, become a key step towards producing hepatocytes [23–25]. The nodal signaling pathway and other important signaling pathways play a major role in this process, including Wnt, PI3K(–), FGF, and BMP signaling pathways. In the processes of cell culture, there are many different combinations of cytokines that regulate these signaling pathways which may give us the opportunity to induce the desired endoderm cells. When inducing the endoderm cells into hepatocytes, the activin/nodal pathway needs to be suppressed, but the Wnt signaling pathway still plays an important role, although it does not require to be exogenously added because definitive endoderm cells abundantly expressed wnt3a [26–28]. In our study, during the endodermal induction step, hADSCs are exposed to a high level of activin/nodal (IDE1), Wnt (CHIR99021), and PI3K(-) (LY294002) signals that are designed to mimic events during embryonic development in order to allow definitive endoderm (DE) formation. IDE1 is a small molecule compound and could induce DE differentiation in up to 50 % of hESCs (or 80 % of mESCs) in the absence of activin A (a typically used DE inducer) [29–32]. During the hepatocyte precursor induction step, we used the cytokines and small molecules which generally start the original intestinal development, such as Wnt (endogenous), activin/nodal (IDE1), BMPs(–) (LDN193189), PI3K(–) (LY294002), and FGF4. In the same way, during the mature hepatocyte induction step, the common cytokines such as FGF4, HGF, OSM, and Dex also simulate the development of the process. Lysophosphatidic acid (LPA) is a multi-function glycerophospholipid which activates β-catenin signaling in stem cells and could be critical for hepatocyte differentiation and survival. It would be interesting to test if LPA added to our induction system could promote generation of iHeps or initiate their self-renewal in vitro. It also would be very useful as iHeps genetically engineered with stable β-catenin may reduce the number of hepatocytes needed for cell transplant therapy and thus eliminate the need for serial transplants [33–35].

In conclusion, our three-step differentiation protocol was capable of inducing hADSCs into hepatocyte-like cells (iHeps) in vitro, which co-expressed ALB and AAT at a high level at the tenth day. iHeps also displayed other functions of mature hepatocytes, such as glycogen storage, ac-LDL, and ICG uptake. Additionally, transplanted iHeps could repopulate livers of mice with acute fulminant liver failure, and restore the liver function. Taken together, these results showed that hADSCs may be a good candidate as well as being ready available from patients’ autologous adipose tissue to treat liver disease. Using the induced hepatocytes (iHeps) as a source of hepatocytes should help the development of alternative methods that may supersede liver transplantation in patients with liver failure. Therefore, hADSCs may be a superior choice for the establishment of a therapy for the injured liver. The possibility for their future autologous transplantation application in the therapy of liver diseases is very promising.

## Conclusions

This present study isolated highly purified hADSCs and provided a new, non-transgenic protocol to induce hADSCs into functional hepatocyte-like cells within 10 days. The results show a very important step towards the future establishment of an alternative and successful therapy using autologous hepatocytes to treat liver disease.

## Abbreviations

AAT, alpha-1 antitrypsin; ADSC, adipose-derived stem cell; AFP, α-fetoprotein; ALB, albumin; ALT, glutamic-pyruvic transaminase; AST, glutamic oxalacetic transaminase; CCl_4_, carbon tetrachloride; CK, cytokeratin; CYP450, cytochrome P450; hADSC, human adipose-derived stem cell; HBSS, Hank’s balanced salt solution; HNF4α, hepatocyte nuclear factor 4α; ICG, indocyanine green; iHep, induced hepatocyte; ITS, insulin-transferrin-selenium supplement; LDL, low-density lipoprotein; MSC, mesenchymal stem cell; OLT, orthotropic liver transplantation; PHH, primary human hepatocyte; TTR, transthyretin
